# Oxidative Stress Statues in Serum and Follicular Fluid of Women with Endometriosis

**DOI:** 10.22074/cellj.2016.4724

**Published:** 2016-09-26

**Authors:** Nahid Nasiri, Ashraf Moini, Poopak Eftekhari-Yazdi, Leila Karimian, Reza Salman-Yazdi, Arezoo Arabipoor

**Affiliations:** 1 Department of Embryology, Reproductive Biomedicine Research Center, Royan Institute for Reproductive Biomedicine, ACECR, Tehran, Iran; 2 Department of Endocrinology and Female Infertility, Reproductive Biomedicine Research Center, Royan Institute for Reproductive Biomedicine, ACECR, Tehran, Iran; 3 Department of Obstetrics and Gynecology, Faculty of Medicine, Tehran University of Medical Sciences, Tehran, Iran; 4 Department of Obstetrics and Gynecology, Arash Women's Hospital, Tehran University of Medical Sciences, Tehran, Iran; 5 Department of Andrology, Reproductive Biomedicine Research Center, Royan Institute for Reproductive Bomedicine, ACECR, Tehran, Iran

**Keywords:** Endometriosis, Follicular Fluid, Lipid Peroxide, Oxidative Stress

## Abstract

**Objective:**

This study aimed to evaluate the levels of two oxidative stress (OS) markers including lipid peroxide (LPO) and total antioxidant capacity (TAC) in both serum and follicular fluid (FF) of women with endometriosis after puncture.

**Materials and Methods:**

In this cross-sectional study, a total number of sixty-three women younger than 40 years old with laparoscopy (gold standard for endometriosis diagnosis) indication underwent *in vitro* fertilization (IVF) program in the Royan Institute, Tehran, Iran from September 2013 to October 2014. About forty-three patients were diagnosed with endometriosis after laparoscopy. Blood and FF from the leading follicle in each stimulated ovary were obtained at the time of egg retrieval; samples were centrifuged and frozen until assessment. At the time of sample assessment, serum and FF samples were evaluated for the levels of LPO and TAC on spectrophotometery.

**Results:**

We observed that women with endometriosis had significantly higher LPO and lower TAC levels in the serum and FF as compared with the control group (P<0.05).

**Conclusion:**

It has observed that FF of women with endometriosis, regardless of disease stage, increases the proliferation power of endometrial cells *in vitro*, we presume that inflammatory reactions-induced OS in ovary may be responsible for proliferation induction ability in FF obtained from women with endometriosis.

## Introduction

Endometriosis as a dynamic complex disorder in about 8-10% of women at reproductive ages is characterized by presence of extra uterine endometrial tissue, dysmenorrhea, pelvic pain and infertility (1,4). Several hypotheses have been proposed to explain its pathogenesis, among which Sampson theory introduced in 1920 indicated the retrograde menstruation through fallopian tube into the peritoneal cavity as the main cause of endometriosis (1). Several studies have reported the elevation of some pro-inflammatory cytokine and growth factor, which could induce inflammation, neoangiogenesis and proliferation of endometriotic implants, in the peritoneal cavity of women with endometriosis (5,6). Ectopic endometrial cell proliferation and chronic inflammation in endometriosis are largely associated with oxidative stress (OS) induction (3,7). OS is a condition in which reactive oxygen species (ROS) overproduction and antioxidant deficiency cause a shift in oxidant/antioxidant balance (2,8). ROS are one of the natural byproducts of the cell metabolism that if they exceed a critical threshold, they cause significant cell structural and functional damages (4). A significant elevation in OS markers concentration, especially lipid peroxides (LPO), has been observed in peritoneal fluid of patients with endometriosis as compared with controls (9,11). However, it has been found that women with endometriosis have low levels of antioxidant in their peritoneal fluid (11,14). In a patient with endometriosis, the residuals from a retrograde bleeding which already existed on the surface of ovaries may enter the ovary and cause the formation of the “chocolate cysts” after invagination of the ovary cortex (15). Furthermore a number of studies have indicated that patients with endometriosis have significantly lower concentrations of some antioxidant components (vitamin A, vitamin C and selenium) in follicular fluid (FF) (12,16), while there are lower concentrations of superoxide dismutase (SOD) and vitamin E in both serum and FF (13,14). FF as a location for reflecting metabolic process that surrounds mature oocyte before ovulation plays a critical role in the reproductive performance of oocyte (17); therefore, endometriosis associated with infertility should be paid a special attention. In present study, we aimed to evaluate the concentrations of two main markers of OS including LPO and total antioxidant capacity (TAC) in both FF and blood serum of patients with endometriosis. We intended to measure the combination of both small molecule antioxidant and protein instead of some antioxidant components (vitamin E, C, and SOD), while we proposed the possible mechanism for the proliferation induction potential of FF. 

## Materials and Methods

### Patient selection

In this cross sectional study, a total number of 63 women younger than 40 years old with laparoscopy (gold standard for endometriosis diagnosis) indication underwent *in vitro* fertilization/intracytoplasmic sperm injection (IVF/ICSI) program at the Royan Institute, Tehran, Iran, between September 2013 and October 2014. Women who had used hormones and antioxidant in the past 3 months were excluded from the study. Patients’ body mass index (BMI), age and smoking status were considered as confounders variables and evaluated by statistical tests. All the participants signed a written informed consent for the collection of blood and FF samples. This study was approved by the Institution Review Board and the local Ethics Committee of Royan Institute, and all data were collected under informed consent and anonymously analyzed (Approval number: EC/90/1046). The patients were divided into 2 groups on the basis of laparoscopy report as follows: group A including 43 women with endometriosis confirmed by laparoscopy and group B including 20 women without macroscopic endometriosis undergoing laparoscopy for other reasons (uterine myoma, tubal infertility and non-endometriotic ovarian cysts) as a control. 

### Stimulation protocol

Standard controlled ovarian stimulation was used for all patients as follows, suppression of pituitary gonadotropin secretion with the gonadotropin-releasing hormone (GnRH) agonist (Suprefact, Hoechst AG, Germany) by subcutaneous (SC) injection (500 mg/d) at the mid luteal phase of the previous ovarian cycle (day 21). After ovarian suppression was confirmed [serum levels of estradiol (E_2_)≤50 pg/ml, follicle-stimulating hormone (FSH)≤12 IU and luteinizing hormone (LH)≤5 IU], ovarian stimulation was initiated by SC injection of 150 IU/day recombinant FSH (Gonal F, Serono, Switzerland). When at least three follicles reached a minimum diameter of 18-20 mm, a single injection of 10,000 IU human chorionic gonadotropin (hCG, Pregnyl, Organon, Netherland) was given. Oocytes were retrieved 36 hours later using a standard ultrasonically guided follicular puncture. 

### Sample collection and processing

The blood samples were collected in 10 ml nonheparinized glass tubes before an intravenous injection of anesthetic agents for puncture of the ovaries. FF of each follicle in all patients was aspirated separately into each tube. FFs from follicles smaller than 15 mm, follicles without egg, follicles with more than 1 oocyte and FF with blood contamination were discarded. The coagulated blood and FF samples were centrifuged at 300 g for 7 minutes to remove cellular remnants, while clear supernatant was frozen at -196˚C (in liquid nitrogen) and kept for up to 1 month before measurements. 

### Determination of total antioxidant capacity in serum and follicular fluid

The sensitive, easy and rapid assay known as ferric reducing/antioxidant power (FRAP) was applied to measure TAC, both in serum and FF (18). This assay is applied to measure the antioxidants as reductants in a redox-linked colorimetric procedure using a spectrophotometer (Bio Aquarius, England). Briefly, 10 mmol/l of 2, 4, 6-tri-(2-pyridyl)-1, 3, 5-triazine 98% (Sigma-Aldrich) (3.1 mg/ ml in 40 mmol/l HCL) and 20 mmol/l FeCl_3_ .6H_2_ O in the ratio of 10: 1: 1 were combined with a 300 mmol/l acetate buffer (pH=3.6), meaning 3.1 g of sodium acetate trihydrate (C_2_H_3_NaO_23_H_2_O) was added to 16 ml of glacial acetic acid that reached to the final volume of 11 ml using distilled H_2_O, as a operant FRAP reagent. Then 50 µl of sample (FF or serum) was added to 1 ml of FRAP reagent in a cuvette. Absorbance was assessed at 593 nm (A_593_) far from direct sunlight and at room temperature by means of 50 µl water as the reference, just 10 minutes after mixing. This means TAC was evaluated as ROS activity. In FRAP assay, standardization was performed using Fe^2+^ concentration, so TAC was equal to the concentration of Fe^2+^ (19). 

### Measurement of lipid peroxide

The concentrations of malondialdehyde (MDA) as an index of LPO in the serum and FF were measured using thiobarbituric acid (TBA) procedure according to study by Das et al. (19). In this method, after reacting MDA with TBA, a red compound with an absorbance at 535 nm was obtained. 

Two ml of stock reagent [12 % (w/v) tricholoro acetic acid. 0.375% (w/v) TBA and 0.25 mol /l warm HCL to dissolve the TBA] was blended with 1 ml of thawed sample (serum or FF), heated in a boiling water bath for 15 minutes and centrifuged for 10 minutes at 1000 g after cooling. Optical density (OD) of supernatant fluid was evaluated against a blank including all the reagents alone. LPO concentration was presented as µM MDA equivalent. 

### Statistical analysis

The obtained result was expressed as mean ± SEM. The statistical analysis was accomplished using the Statistical Package for the Social Sciences (SPSS, SPSS Inc., USA) version 16. Inter group differences were assessed with student’s t test. Normality of data was checked by Kolmogorov-Smirnov test. Statistical significance was defined by P<0.05. 

### Results

**Fig.1:**
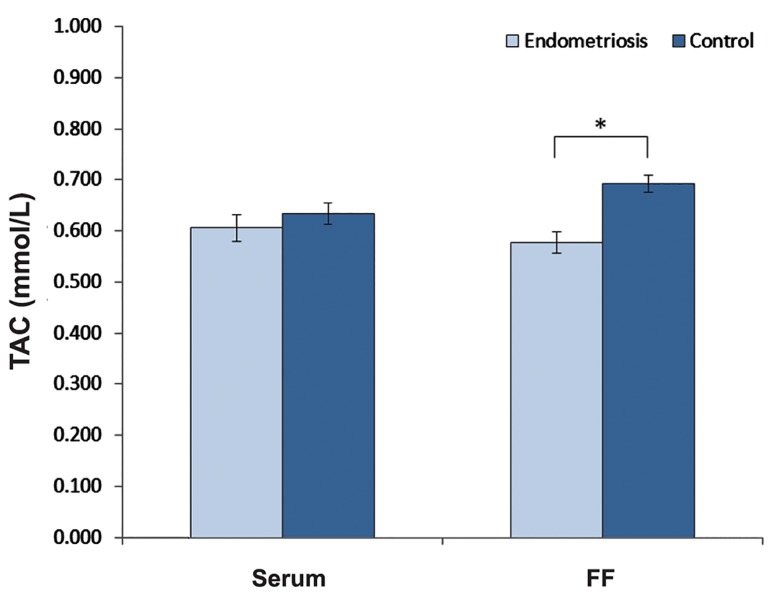
TAC concentration in serum and FF of endometriosis and control women. Data are presented as mean ± SEM. Statistical significance level is P<0.05. TAC; Total antioxidant capacity and FF; Follicular fluid.

Figure 1 demonstrates the serum level of TAC in serum and FF of two groups. The concentration of TAC in FF of women with endometriosis was significantly lower than those in control group (0.577 ± 0.018 vs. 0.692 ± 0.017, P=0.03). No significant difference was observed in serum level of TAC between two groups (P>0.05) and no correlations were noted for TAC concentration between serum and FF contents of each group. 

**Fig.2:**
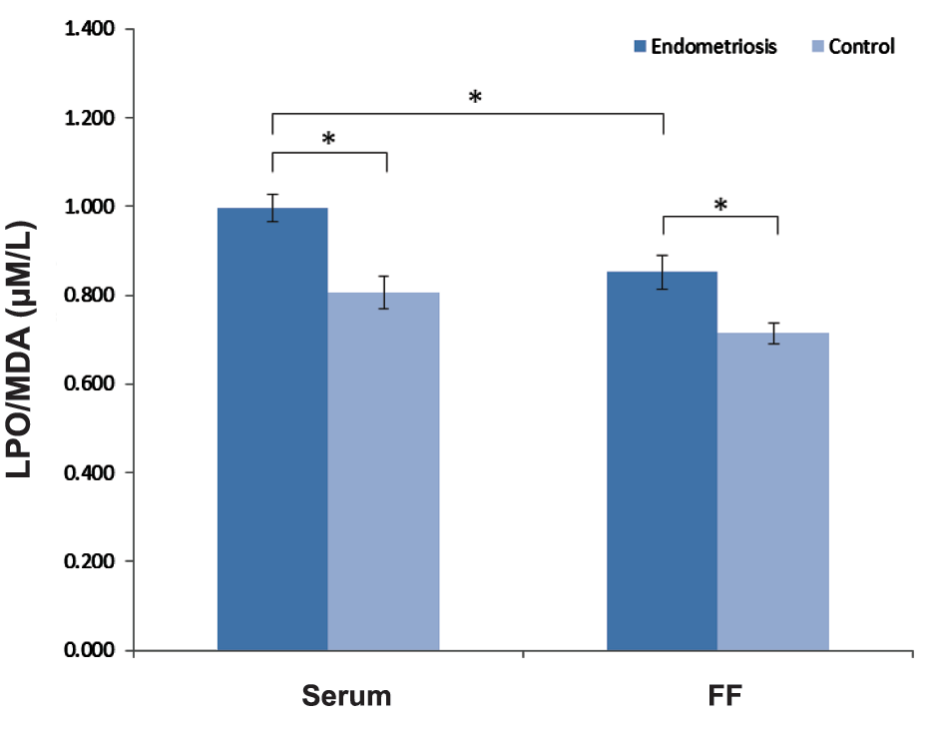
LPO concentration in serum and FF of endometriosis and control women. Data are presented as mean ± SEM. Statistical significance level is P<0.05. FF; Follicular fluid and LPO; Lipid peroxide.

Figure 2 illustrates the LPO levels in serum and FF samples of two groups. Our result demonstrates a significant increase in LPO levels of serum and FF in endometriosis patients as compared with control women (P<0.05). Moreover, the serum content of endometriosis women had significantly higher concentration of LPO in comparison with FF of these patients (0.997 ± 0.03 vs. 0.852 ± 0.04, P=0.02). 

## Discussion

Results of this study demonstrated that the FF and blood serum of women with endometriosis has significantly higher levels of LPO and lower level of TAC as compared with women in control group. This result is in accordance with several prior studies which have reported the elevated levels of OS markers and reduced levels of TAC in serum and FF of women with endometriosis (12,14). In a study by Murphy et al. (16), they have reported the presence of oxidatively modified lipid-protein complexes in endometrium and endometriosis tissues. In two recent studies by Singh et al. (12) and Liu et al. (13), they have reported lower concentrations of some antioxidant components in FF alone (12) and in both serum and FF (13,14) of patients with endometriosis as compared with control normal women. 

Recently, the impact of OS on infertility has been evaluated because OS is likely to solve the mystery of some infertility cases such as endometriosis (20). Many studies have reported the close relationship between OS and the following factors: endometriosis (10,20), activated macrophages, growth factors, cytokines, and undigested or apoptotic endometrial cells remained from retrograde menstruation in peritoneal fluid. These factors are found to be elevated in patients with endometriosis (2,4) that leads to induce OS (11). Most of these studies have focused on the OS induction in peritoneal fluid, so there is a lack of study on follicular fluid. It is possible that FF provides a suitable environment for aggregation and growth of endometriotic cells; hence, it might play an important role in pathogenesis of endometriosis (20). FF as one of the components of peritoneal fluid after ovulation may contain the growth-promoting factors that transfer them into the peritoneal cavity in endometriosis patients (21). 

It is observed that endometriosis is one of the factors inducing ovarian cancer that is associated with ovarian inflammation (22). Induced inflammation in ovary may increase the concentrations of cytokine and other pro-inflammatory factors that lead to induction of intra ovarian OS (2,7). Bahtiyar et al. (21) have demonstrated that FF collected from patients with endometriosis is able to induce the endometrial cell proliferation higher than FF from those women without endometriosis and this potency is independent of endometriosis levels (minimal-mild or moderate-sever). On the other hand, it is observed that OS induction is associated with increased proliferation rate of endometrial cells as well as tumor cells, meaning ROS is known as a second messenger of cell proliferation (23). Based on these observations, it seems that OS induction in FF of women with endometriosis is responsible for inducing a proliferation response observed in FF of these patients which its reason was previously unknown. FF may carry the induced-OS into the peritoneal fluid after ovulation that lead to increased proliferative phenotype of ectopic endometrial cells (3). 

In current study, in addition to increased level of LPO, it was also observed that the concentration of TAC in FF of women with endometriosis was significantly lower than those in control group. Furthermore, the antioxidants aggregate cumulative action and synergic effect were evaluated thorough measuring TAC (combination of both small molecule antioxidant and protein). Similarly, in a study by Szczepańska et al. (9), they have indicated OS-induction in patients with endometriosis. Also, they have showed that a decrease in TAC concentration led to an increase in ROS concentration in endometriosis women (24). 

A comparison between serum and FF showed that the LPO concentration in serum is greater than FF, especially in endometriosis patients, suggesting that there is a potential antioxidant support inside the preovulatory FF (19) that is likely due to accumulation of estrogens (25) in FF. On the other hand, the lower level of LPO in FF as compared to the related value in serum may be representative for the absence of free exchange of lipoprotein between the FF and serum (26). 

Laparoscopy -as an invasive diagnostic techniqueis still the gold standard for diagnosis of endometriosis; therefore, evaluation of biomarkers, especially ROS and OS levels, is suggested in patient with endometriosis, mainly for identification of non-invasive diagnostic method. The results of present study confirmed the observations of previous reports (12,16), so we suggested that the ROS biomarkers will be evaluated as non-invasive diagnostic markers for endometriosis. Owing to key role of OS in pathogenesis of endometriosis, it seems that antioxidant prescription may improve symptoms as many studies have reported the recuperative effects of antioxidant therapy in these patients (8,27). However, such evaluations requires more well-designed human studies with large sample size which were the main limitation of our study, indicating that these types of studies are the prerequisites for identification of accurate non-invasive method to diagnosis of endometriosis. 

## Conclusion

Our results suggested that FF of women with endometriosis, regardless of disease stage, increases the proliferation potential of endometrial cells *in vitro*. We presumed that inflammatory reactionsinduced OS in ovary may be responsible for proliferation induction ability in FF obtained from women with endometriosis. 
